# Restoration of T cell tolerance in primary ITP

**DOI:** 10.1186/1756-8722-5-S1-A5

**Published:** 2012-04-25

**Authors:** Xin-guang Liu, Jun Peng, Ming Hou

**Affiliations:** 1 Department of Hematology, Qilu Hospital, Shandong University, 107 West Wenhua Road, Jinan, P. R. China

## 

Primary immune thrombocytopenia (ITP) has been traditionally thought as an antibody-mediated autoimmune disease involving platelet destruction by macrophages in the reticuloendothelia system. More recently it has become obvious that ITP is a more complex disorder in which T cell mediated immunity plays important roles in platelet destruction. Antiplatelet autoantibody production is under the control of platelet-specific helper T-cells, and loss of tolerance to self antigen by T cells is the critical step of the immune dysregulation in ITP. Dendritic cells (DCs) from ITP patients showed enhanced capacity in stimulating autologous T-cell proliferation in the presence of autologous/allogeneic platelets [1], and ITP patients’ T cells had elevated IL-2 secretion ability compared with controls [2,3], suggesting increased antiplaltelet T-cell reactivity in ITP. The epitopes that recognize platelet glycoprotein (GP) IIIa on T helper (Th) cells has been determined and mapped by several groups [4,5], thus sheding new lights on the “therapeutic vaccination” approach to reinstate tolerance in ITP. Autoreactive T-cell reactivity against platelet antigen in active ITP patients has been observed at polyclonal as well as oligoclonal levels [6,7]. Our group has demonstrated that blocking the B7-CD28 interaction with CTLA4-Ig/CsA could induce platelet GP-specific T-cell anergy, which could exert suppressive effect on GP-reactive T cells via inducing tolerogenic dendritic cells (DCs) [8,9]. It has been well established that apoptotic genes, such as Fas, A20, Bax, Calpastatin, IL2RB, were expressed aberrantly in patients with active ITP [10,11], leading to autoreactive T cells resistant to activation induced cell death (AICD), which could in turn support the expansion of self-reactive T-cell clones. A loss of resistant to AICD might be an important mechanism for the achievement of remission in ITP. Previous studies have revealed that dexamethasone could suppress T-cell proliferation and induce apoptosis of T-cells in ITP [11,12]. In addition, our group has demonstrated that a novel BAFF blocking reagent, BR3-Fc, could restore the apoptosis of both B and T cells [13]. Th polarization in ITP has been attributed to increased Th1 [2,14], and Th17 cells [15] or reduced number or function of CD4^+^CD25^+^Foxp3^+^ T-regulatory cells (Tregs) [16,17]. A parallel body of aberrant cytokine patterns, such as the elevated ratio of interleukin (IL) -18/IL-18 binding protein (BP) [18,19], the increased expression of B cell activating factor (BAFF) has been reported in active ITP patients [20,21]. High-dose dexamthasone (HD-DXM) could not only restore Th1/Th2 [14] or IL-18/IL-18BP balance [19], but also increase the number of Tregs [16], and inhibit the expression of BAFF [12]. Besides HD-DXM, multiple agents, such as rituximab [22], intravenous immunoglobulin (IVIg) [23], romiplostim, eltrombopag [24] as well as indirubin [25], could increase the number or restore the function of Tregs in ITP. Our recently study showed that GP-specific induced Tregs could be successfully generated de novo from nonregulatory CD4^+^CD25^-^CD45RA^+^ cells and could mediate both antigen-specific and linked suppression of proliferating antiplatelet CD4^+^ Th cells in vitro, and further research revealed that the de novo expanded Tregs mediated their suppressive effects on T cells via actually modulating the T-cell stimulatory capacity of DCs [26], thus providing a clue to the potential of producing antigen-specific Tregs from the patients in vitro for the purpose of antigen-targeted cellular immunotherapy. In conclusion, induction of T-cell tolerance may provide a useful strategy for the management of ITP.
